# Significant contribution of metastable particulate organic matter to natural formation of silver nanoparticles in soils

**DOI:** 10.1038/s41467-019-11643-6

**Published:** 2019-08-21

**Authors:** Ying-Nan Huang, Ting-Ting Qian, Fei Dang, Yong-Guang Yin, Min Li, Dong-Mei Zhou

**Affiliations:** 1 Key Laboratory of Soil Environment and Pollution Remediation, Institute of Soil Science, Chinese Academy of Sciences, 210008 Nanjing, People’s Republic of China; 2 University of Chinese Academy of Sciences, 100049 Beijing, People’s Republic of China; 3 State Key Laboratory of Environmental Chemistry and Ecotoxicology, Research Center for Eco-Environmental Sciences, Chinese Academy of Sciences, 100085 Beijing, People’s Republic of China; 4 Present Address: Advanced Environmental Biotechnology Centre, Nanyang Environment and Water Research Institute, Nanyang Technological University, 1 Cleantech Loop, Singapore, 637141 Singapore

**Keywords:** Element cycles, Environmental sciences, Environmental chemistry

## Abstract

Particulate organic matter (POM) is distributed worldwide in high abundance. Although insoluble, it could serve as a redox mediator for microbial reductive dehalogenation and mineral transformation. Quantitative information on the role of POM in the natural occurrence of silver nanoparticles (AgNPs) is lacking, but is needed to re-evaluate the sources of AgNPs in soils, which are commonly considered to derive from anthropogenic inputs. Here we demonstrate that POM reduces silver ions to AgNPs under solar irradiation, by producing superoxide radicals from phenol-like groups. The contribution of POM to the naturally occurring AgNPs is estimated to be 11–31%. By providing fresh insight into the sources of AgNPs in soils, our study facilitates unbiased assessments of the fate and impacts of anthropogenic AgNPs. Moreover, the reducing role of POM is likely widespread within surface environments and is expected to significantly influence the biogeochemical cycling of Ag and other contaminants that are reactive towards phenol-like groups.

## Abstract (web summary)

Silver nanoparticles are known environmental contaminants, however it is unclear whether they arise in soils through natural processes, anthropogenic processes, or both. Here Huang and colleagues offer fresh insight into the natural formation of these contaminants by soil particulate organic matter exposed to solar irradiation.

## Introduction

Soil organic matter, a continuum of progressively decomposing organic compounds, plays an important role in the global carbon budget and nutrient retention^1,2^. Most organic matter in soils is in particulate, known as humin, rather than in a dissolved form^3^. Particulate organic matter (POM) has been identified as a metastable phase^4,5^, i.e., insoluble but could be chemically active in extracellular electron transfer in microbial reduction of, for example, iron oxide and pentachlorophenol dechlorination^6–8^. However, the effects of POM on the geochemical transformation of trace metals are unclear. Considering that POM is distributed worldwide in high abundance^3,5^, typically comprising >50% of soil organic matter in mineral soils and >70% of that in lithified sediments^4^, identifying the mechanisms underlying its reactivity will provide new insight into its role in the cycling of numerous trace metals within surface environments.

One of the current major concerns is the unintentional release of anthropogenic silver nanoparticles (AgNPs), which have unique properties, leading to specific benefits and impacts^9^. Recently a significant fraction of AgNPs has been directly detected in ionic silver-contaminated soils, where anthropogenic input (e.g., anthropogenic AgNPs in industrial, medical, and consumer products) was unlikely^10–13^. Therefore, this work aimed to reveal the formation processes and mechanisms of naturally occurring AgNPs in soils. Although silver ions (Ag^+^) can be readily reduced to AgNPs by microorganisms and their associated extracellular polymeric substances^14–16^, the mechanism allowing the abiotic reduction of Ag^+^ is largely unknown. Studies on the role of organic matter in natural formation of AgNPs have focused on dissolved organic matter (DOM) in lakes and rivers^17–20^, but an extrapolation of the results to POM is difficult due to the distinct differences in the physical and the chemical properties of these two media^4,5,21^. Therefore, DOM fails to explain the natural formation of AgNPs in soils^17,19^. The higher relative abundance of POM than DOM and the redox ability of POM lead us to hypothesize that POM participates in reducing Ag^+^ to AgNPs at the soil surface. A demonstration of this interaction would be of considerable importance, as it would enable researchers to re-evaluate the presence and source of AgNPs in terrestrial environment, and thus to conduct unbiased assessments of fate and impacts of anthropogenic AgNPs.

In this work, we identify a clear role for POM in the natural formation of AgNPs, both in the sand matrix and heterogeneous suspensions. The use of this simplified system has significant advantages over natural samples, as we are able to investigate the specific chemical mechanisms, largely without biotic interferences. The molecular mechanism of POM-mediated AgNP formation was analyzed using a novel combination of electron paramagnetic resonance (EPR), solid-state ^13^C nuclear magnetic resonance (NMR), and fourier-transform infrared (FTIR) spectroscopy. The resulting mechanistic insights into the interactions between POM and Ag will improve our current understanding of the geochemistry of trace metals in soils.

## Results

### Reduction in sand matrix

**Fig. 1:**
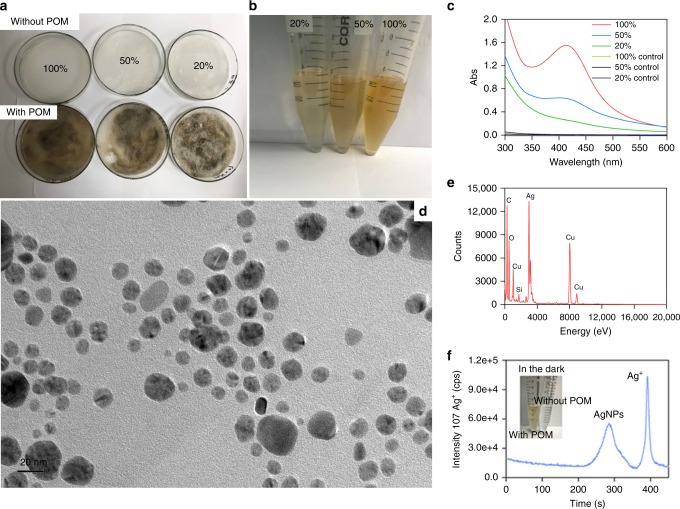
Silver nanoparticles in sand matrix under natural irradiation. **a** Representative sand matrix samples without or with particulate organic matter (POM) at 20%, 50%, and 100% humidity under natural irradiation for 10 h at ~30 °C. **b** Color evolution of samples collected from the sand matrix in the presence of POM under conditions of different humidity. **c** Characteristic absorbance spectrum of AgNPs recorded by UV–Vis spectra in the supernatants. **d** TEM image of AgNPs formed in supernatants of samples with water-holding capacity of 100% in **a**. **e** EDS analysis of particles in **d**. **f** The formation of AgNPs after interaction of Ag^+^ and POM in sand matrix in the dark as revealed by LC-ICP-MS

The natural formation of AgNPs at soil surface was mimicked with a sand depth of 1 mm (light penetration in natural soils is 0.2−0.4 mm)^22^ and exposure of the samples to natural sunlight irradiation for 10 h outdoors (Fig. 1a). In the presence of POM, the color of the supernatants was light brown (Fig. 1b). The formation of AgNPs was confirmed by the characteristic peak of surface plasmon resonance (SPR) at ~400 nm on ultraviolet (UV)–visible (Vis) spectrometry^18^ (Fig. 1c). A notable amount of AgNPs with diameters of 12.8 ± 4.5 nm was observed in the liquid phase by transmission electron microscopy (TEM) with energy dispersive X-ray spectrometry (EDS) (Fig. 1d, e). POM not only facilitated the formation of AgNPs in the presence of light, it also worked in the dark, as detected by liquid chromatography inductively coupled plasma mass spectrometry (LC-ICP-MS) (Fig. 1f). No AgNPs formed in the irradiated control without POM. These results indicated a significant role of POM in AgNPs formation at soil surface, despite the limited penetration of sunlight in the soil.

### Reduction in simplified suspension

Experiments were then performed by incubating 9.3 × 10^−3^−0.93 mM Ag^+^ with POM at 9.0−143.2 mg C L^−1^ under natural sunlight. After 24 h, the suspensions were pale yellow (Supplementary Fig. 1) but only the suspension containing 0.93 mM Ag^+^ had an obvious SPR absorbance at ~400 nm (Supplementary Fig. 2b). Further, the intensity of SPR at ~400 nm increased with increasing POM concentrations (Supplementary Fig. 2b).

**Fig. 2:**
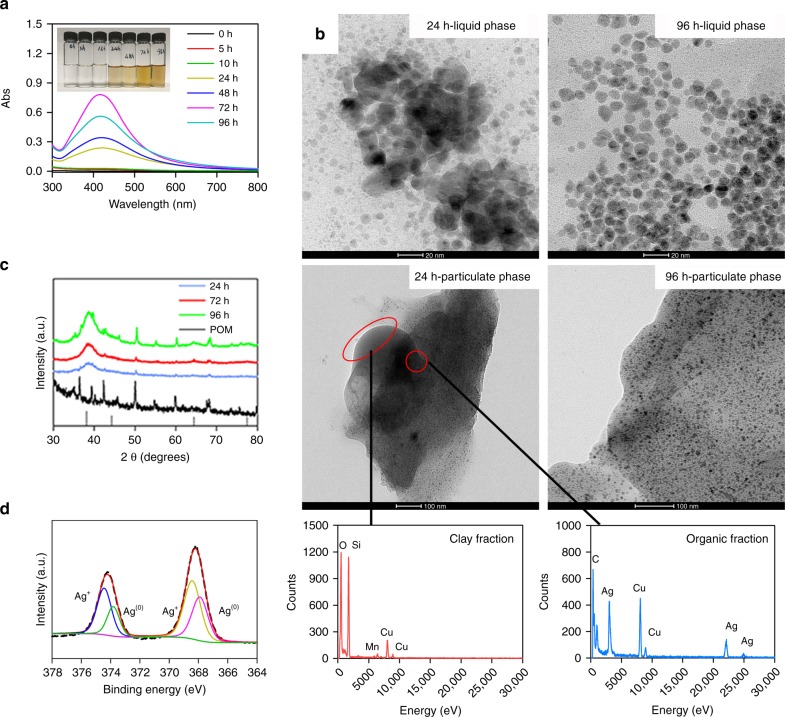
Silver nanoparticles in simplified suspension under simulated sunlight. Silver ions (Ag^+^) at 0.93 mM were incubated with particular organic matter (POM) at 143.2 mg C L^−1^ at pH 8.6. **a** UV–Vis spectra of AgNPs in liquid phase (insert showing the color evolution of the sample over time). **b** TEM images of AgNPs in liquid and particulate phase and the respective EDS analysis after 24 h of incubation with POM. **c** XRD analysis of Ag loaded onto POM. **d** XPS analysis after the incubation of Ag^+^ with POM for 24 h

To further explore the operative conditions favoring Ag^+^ reduction, 0.93 mM Ag^+^ was allowed to react with 143.2 mg C L^−1^ POM under simulated sunlight over pH 5.6−8.6 at 25 °C (Fig. 2a and Supplementary Fig. 3c). After 24 h, all suspensions were yellow but only those under neutral and alkaline conditions (7.0−8.6) had obvious SPR absorbance at ~400 nm (Supplementary Fig. 3c). This is attributed to the low dissolution of AgNPs^23^ and an increasing trend of radical signal intensity at higher pHs (as discussed later). Figure 2b shows typical TEM images of nanoparticles at pH 8.6, where notable amounts of nanoparticles with average diameters of 8.4 ± 3.7 and 7.7 ± 3.8 nm were observed in liquid and particulate phase after 24 or 96 h incubation; the size was similar to that of samples from sand matrix (Fig. 1d). EDS confirmed the presence of Ag (Fig. 2b). On X-ray powder diffraction (XRD) analysis, the strong diffraction peak occurring at 38.2° in the POM corresponded to metallic Ag facets of (111) (Fig. 2c). The species of Ag was further confirmed by X-ray photoelectron spectroscopy (XPS) analysis. The signals of Ag 3d_3/2_ and 3d_5/2_ were located at 373.9 eV and 367.9 eV (Fig. 2d), characteristic of metallic Ag^24^. AgNPs did not form in Ag^+^ solution in the absence of POM (Supplementary Fig. 3a). Combined, the UV–Vis, TEM-EDS, XRD, and XPS results provides conclusive evidence of the formation of metallic AgNPs in the presence of POM under irradiation for 24 h.

The possible role of DOM released from POM in reducing Ag^+^ to AgNPs was minimal based on the following evidence. Firstly, very little AgNPs formed when POM was replaced with its released DOM (Supplementary Fig. 4a, b). Secondly, the EPR signal of ${\mathrm{O}}_2^{\mathrm{\cdot-}}$ was not detected in the released DOM (Supplementary Fig. 4c, as described later). Finally, AgNPs were formed in a dialysis bag (pore size < 1 nm, Spectrum, USA) containing POM but not in an incubation consisting of a bulk solution and released DOM (Supplementary Fig. 5). These observations suggest that, at least in our experimental system, POM serves as a natural insoluble agent for the reduction of Ag^+^ to AgNPs under irradiation.

### Reduction kinetics

**Fig. 3:**
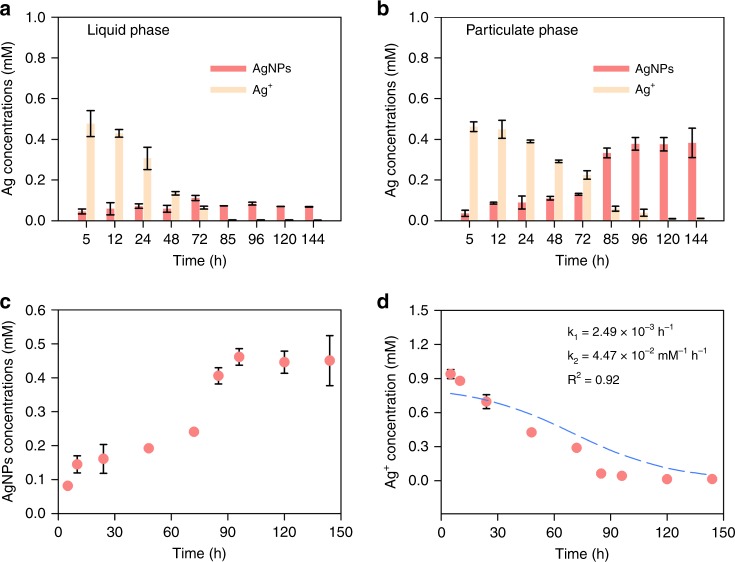
Kinetics of silver nanoparticles formation. Silver ions (Ag^+^) at 0.93 mM were incubated with particular organic matter (POM) at 143.2 mg C L^−1^ under simulated sunlight at pH 8.6. The amount of AgNPs and Ag^+^ in **a** liquid and **b** particulate phase. Total amount of **c** AgNPs and **d** Ag^+^ in the heterogeneous system, and the experimental data were fitted using the Finke–Watzky model. Error bars represent the standard deviation of the mean of the triplicates

In the heterogeneous system, only ~15% of total AgNPs were distributed in liquid phase and the concentration was relatively constant after 85 h of incubation (~0.06 mM, Fig. 3a, and Supplementary Fig. 6a, c). By contrast, the amount of AgNPs in the particulate phase increased continuously (Fig. 3b and Supplementary Fig. 6b, d) and accounted for up to ~85% of the total AgNPs formed (Fig. 3b).

The kinetics of AgNPs formation by POM were best described by the Finke–Watzky model that involves two pseudoelementary steps^25^, by which Ag^+^ was first reduced to zerovalent atoms (nuclei formation, ${\mathrm{{Ag}}}^ + + {\mathrm{{e}}}^ - \mathop{\longrightarrow}\limits^{k_{\mathrm{1}}} {\mathrm{{Ag}}}^0$) for their aggregation into nuclei $\left( {{\mathrm{Ag}}_n^0} \right)$, followed by autocatalytic surface reduction enabled by the surface sites from just-formed nuclei of ${\mathrm{Ag}}_n^0$ (surface autocatalytic reduction, ${\mathrm{{Ag}}}_{\mathit{n}}^0 + {\mathrm{{Ag}}}^ + + {\mathrm{{e}}}^ - \mathop{\longrightarrow}\limits^{k_{2}} {\mathrm{{Ag}}}^0_{n + 1}$). The autocatalytic reduction of salt precursor is also involved in the synthesis of other metal nanocrystals^26,27^. Therefore, the AgNPs formation could be expressed as^25^:

1 $$\frac{{\mathrm{d}\left[ {{\mathrm{Ag}}_{n}^0} \right]}}{{\mathrm{d}}t} = - \frac{{\mathrm{d}\left[ {{\mathrm{Ag}}^ + } \right]}}{{{\mathrm{d}}t}} = k_1\left[ {\mathrm{Ag}}^ + \right] + k_2\left[ {\mathrm{Ag}}^ + \right]\left[ {{\mathrm{Ag}}_n^0} \right]$$

2 $$\left[ {\mathrm{Ag}}_{{n}}^0 \right] = \left[ {\mathrm{Ag}^ + } \right]_0 - \left[ {\mathrm{Ag}^ + } \right]$$

where $\left[ {\mathrm{Ag}_n^0} \right]$ and [Ag^*+*^] are the concentrations of AgNPs and Ag^+^ in the heterogeneous system at time *t*, respectively; [Ag^+^]_*0*_ is the concentrations of Ag^+^ initially. The *k*_*1*_ and *k*_*2*_ are the rate constants for nuclei formation and surface autocatalytic reduction, respectively. *k*_*1*_ and *k*_*2*_ were derived from curve fitting according the Finke–Watzky model (Fig. 3d)^25–27^, and were 2.49 × 10^−3^ h^−1^ and 4.47 × 10^−2^ mM^−1^ h^−1^, respectively. Thus, the nuclei formation was rate limiting (*k*_*1*_ ≪ *k*_*2*_). The reduction rates of nuclei formation and autocatalysis surface reduction were further modeled as a function of reaction time (Supplementary Fig. 7). The autocatalytic reduction rate increased rapidly over time and after ~7 h was higher than the rate of nuclei formation, suggesting that nuclei formation surpasses nuclei formation to become the dominant process. The rate constant *k*_*1*_ for POM *(k*_*1*_ = 2.49 × 10^−3^ h^−1^) was roughly similar to that in DOM (1 × 10^−3^−0.3 h^−1^)^20,28,29^, suggesting that POM could reduce Ag^+^ at comparable rates to DOM in lakes and rivers. However, the Finke–Watzky two-step mechanism of AgNPs formation by POM was not analogous to DOM with one-step mechanism^20,28,29^. A mechanistic understanding of the autocatalytic surface reduction was not the main focus of this study but merits further investigation. Note that the oxidation of AgNPs was not included in this model because oxidation was not favored under alkaline conditions, based on a reported pseudo-first-order rate constant of 9.58 × 10^−4^ h^−1^^30^.

### POM induced superoxide reduction of Ag^+^

**Fig. 4:**
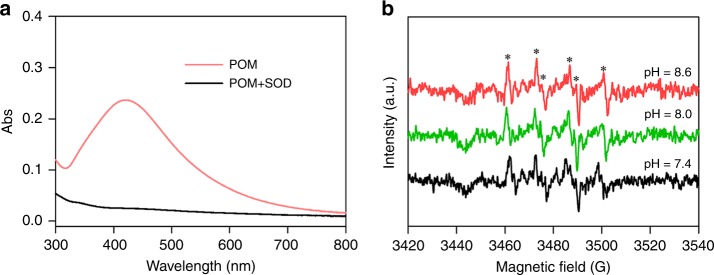
Production of ${\mathrm{O}}_2^{\mathrm{\cdot-}}$ in particulate organic matter suspension. **a** A dramatic decrease in the SPR absorbance of the AgNPs suspension in the presence of 150 U SOD mL^−1^, produced by the reduction of 0.93 mM silver ions (Ag^+^) with 143.2 mg C L^−1^ particulate organic matter (POM) at pH 8.6 under simulated sunlight for 24 h. **b** EPR spectra of the DMPO adduct with ${\mathrm{O}}_2^{\mathrm{\cdot-}}$ in POM suspension at different pHs after incubation of the samples under simulated sunlight for 1 h

The formation of AgNPs could be facilitated under irradiation, under highly alkaline conditions, or in the presence of dissolved O_2_ (Supplementary Fig. 3). The stimulated effect of dissolved O_2_ on AgNPs formation (Supplementary Fig. 3b) indicates that reactive oxygen species (ROS) are responsible for the observed reduction^31^. The ROS mediating AgNPs formation was identified as superoxide ${\mathrm{O}}_2^{\mathrm{\cdot-}}$ because the addition of superoxide dismutase (superoxide scavenger)^18^ in the suspensions abolished AgNPs formation (Fig. 4a). This result is consistent with the suggested role of aquatic derived-DOM in promoting reduction of Ag^+^ under illumination^18^.

**Fig. 5:**
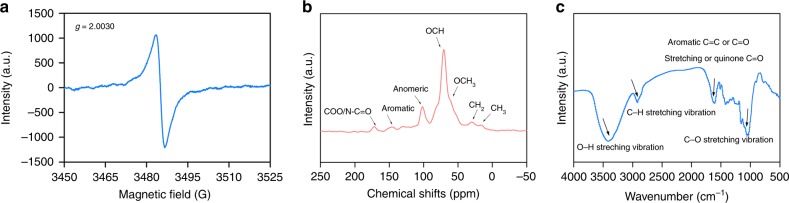
Characteristics of particular organic matter. **a** EPR spectrum. **b** ^13^C NMR spectrum. **c** FTIR spectrum

EPR spectroscopy coupled with 5,5-dimethyl-1-pyrroline-N-oxide (DMPO) as a spin-trapping agent confirmed the generation of superoxide. As illustrated in Fig. 4b, six characteristic peaks of the ${\mathrm{DMPO}} - {\mathrm{O}}_2^{\mathrm{\cdot-}}$ spin adducts^32^ were observed in the POM suspension under simulated sunlight irradiation over a pH range from 7.4 to 8.6. By contrast, the EPR signal of ${\mathrm{O}}_2^{\mathrm{\cdot-}}$ was not detected in the DOM released from POM (Supplementary Fig. 4c); the ${\mathrm{O}}_2^{\mathrm{\cdot-}}$ was thus not directly produced from DOM. Further, EPR spectra of POM in the absence of DMPO was corrected (Fig. 5a), and a single peak was observed with *g* factor value of 2.0030. The *g* factor value is characteristics of semiquinone radicals^33,34^, and thus suggests that semiquinone radicals are produced in the POM. It has been well established that semiquinone radicals produced from quinone-hydroquinone moieties can mediate the formation of ${\mathrm{O}}_2^{\mathrm{\cdot-}}$ via single-electron transfer process with oxygen^35^. Therefore, the electron-donating phenol-like groups of POM (e.g., hydroquinones) are likely responsible for ${\mathrm{O}}_2^{\mathrm{\cdot-}}$ generation. In contrast, no formation of AgNPs was observed in the reaction between ash and Ag^+^; intact and de-ashed POM showed comparable SPR absorbance intensity of AgNPs (Supplementary Fig. 4d). The radicals are thus not directly influenced by clays in POM.

^13^C NMR and FTIR analyses were performed to further identify the organic moieties in POM that were responsible for ${\mathrm{O}}_2^{\mathrm{\cdot-}}$ production under illumination. The ^13^C NMR spectrum confirmed the existence of phenolic moieties (Fig. 5b and Supplementary Table 1). Furthermore, on the FTIR spectrum, the peak of pristine POM occurred at ~3400 cm^−1^, which was attributed to the O–H vibration of the carboxylic and alcoholic groups while the peak at ~1600 cm^−1^ was assigned to aromatic C = C or C = O stretching and quinone C = O^36,37^. The peaks at ~2917 cm^−1^ and 1000 cm^−1^ were assigned to the C–H stretching motions of aliphatic groups and the C–O stretching of polysaccharide, respectively (Fig. 5c)^36^. Collectively, our data points toward that phenol-like groups are important redox-active functional groups in POM, which could be excited under illumination, thus transferring electrons to dissolved O_2_ to form superoxide radicals^37,38^. Additionally, it is reported that the presence of particulates could increase the reactivity of superoxides^39^, which may explain why more AgNPs formed in particulates relative to liquid phase (Fig. 3a, b). The role of particulates in soil matrix in formation/decaying of superoxide awaits further investigation.

## Discussion

Our hypothesis that POM is redox active is supported by all our experimental results. POM reduces Ag^+^ to AgNPs under irradiation and in the dark, suggesting the photoreduction and non-photoreduction pathway. EPR, ^13^C NMR, and FTIR analyses reveal that phenol-like groups are involved in the redox activity of POM (Fig. 5). The redox potentials of these groups^40,41^ and POM^7,36^ are documented to range from –0.49 to 0.37 V (versus a standard hydrogen electrode) and for Ag^+^ is 0.8 V (versus a standard hydrogen electrode)^42^. These result in negative reaction free energies, ranging from −124.5 to −41.5 kJ mol^−1^. Reduction of Ag^+^ by POM is, thus, thermodynamically favorable. The photoreduction pathway was predominately mediated by ${\mathrm{O}}_2^{\mathrm{\cdot-}}$, as evidenced by the EPR signal of ${\mathrm{O}}_2^{\mathrm{\cdot-}}$ (Fig. 4b). The non-photoreduction pathway could be attributed to direct electron transfer from phenol-like groups within POM to Ag^+^. However, there was insufficient amounts of AgNPs in the dark, as reflected by little or no change in the SPR absorbance at ~400 nm by UV–Vis spectrometry (Supplementary Fig. 2a) or diffraction peaks representative of metallic Ag by XRD (Supplementary Fig. 2c).

There are two possible mechanisms of natural AgNPs formation at soil surface: abiotic reduction via POM as shown in this study and biotic reduction (e.g., microbacteria and their associated extracellular polymer substances)^14,43^. The relative importance of these sources to AgNPs formation in soils has not yet been quantified. Typically, Ag concentrations in soils ranges from 0.01 to 126.0 mg kg^−1^ (9.3 × 10^−5^–1.2 mM)^10,44^. Based on an extrapolation of our results to these environmental relevant concentrations, we estimate that between 1.2 × 10^−3^ and 53.0 mg AgNPs kg^−1^ would be formed from abiotic reduction via POM at equilibrium (based on *k*_*1*_ = 2.49 × 10^−3^ h^−1^ and *k*_*2* = _4.47 × 10^−2^ mM^−1 ^h^−1^). In contrast, the biotic reduction rate of AgNPs is 0.056 h^−1^ (ref. ^29^), which should yield from 9.3 × 10^−3^ to 117.4 mg AgNPs kg^−1^ (obtained from pseudo-first-order kinetic)^29^. Thus, 11–31% of the AgNPs in the soil surface may originate from POM, as compared to 69–89% of biotic reduction. This may reflect high AgNP concentrations in POM-rich soils or sediments. This study clearly demonstrates that POM can contribute significantly to the natural formation of AgNPs in soils.

Taken together, our results introduce a new pathway for AgNPs formation in soils, whereby ${\mathrm{O}}_2^{\mathrm{\cdot-}}$, generated from phenol-like groups within POM under irradiation, is a key determinant of Ag^+^ reduction. Even in the dark, POM is redox active in AgNPs formation. Consequently, these results highlight the importance but unrecognized role of POM in naturally occurring AgNPs in soils. Given POM is associated with iron oxides, clays and DOM in natural soils and sediments^3–5^, the formation of AgNPs could be more complex in nature. This reductive pathway will ultimately raise the concerns of POM in the biogeochemistry of contaminants that are reactive towards phenol-like groups.

## Methods

### Extraction and characterization of POM

POM was extracted from a peat soil with 34.1% organic carbon from Changbai Mountain, China (42°9′51″N, 126°44′7″E). Briefly, the air-dried and sieved soil (0.2 mm) was progressively extracted with 0.1 M Na_4_P_2_O_7_ eight times, 0.1 M NaOH 20 times, and then 0.2 M NaOH ten times with an extractant/soil ratio of 10:1, followed by centrifugation at 4500 × *g* for 20 min. The samples were then washed with Milli-Q water, freeze-dried, ground until they were fine enough to pass through a 100-mesh (0.15 mm) sieve^45^ and used in the analyses described below.

The carbon, hydrogen, nitrogen, and oxygen contents of the POM were determined using a Vario EL III element analyzer (Germany) (Supplementary Table 1). The ash content was determined by heating the POM sample at 800 °C for 4 h and calculated based on the mass difference (Supplementary Table 1)^46^. Subsamples were de-ashed in 1.6 M HCl and 3 M HF at extractant/soil ratio of 10:1 for 24 h seven times (designated as de-ashed POM). DOM released from the POM suspension was monitored over 96 h using a total organic carbon analyzer (Multi N/C 3100, Analytik, Jena, Germany).

### Reduction in sand matrix

The experiment was performed to mimic the possible natural formation of AgNPs in the presence of POM on the soil surface. Commercial quartz sand with a grain size of 0.3−0.7 mm was thoroughly cleaned with 0.01 M HNO_3_ and NaOH^47^. A uniform layer of 1 mm sand was formed in glass Petri dishes (9 cm in diameter) using 14 g of quartz sand, with or without 0.3 g of POM, corresponding to POM content in natural soil^48^. Each experimental group was spiked with Ag^+^ (as AgNO_3_, pH 8.6) at 100 mg kg^−1^ (dry weight), rewetted periodically to maintain a water-holding capacity of 20, 50, and 100%, covered with polyvinyl chloride film, and irradiated for 10 h outdoors under natural sunlight [6,820–178,900 lux, measured using a digital lux meter (BENETECH GM1010, China)]. After 10 h, the resulting AgNPs were analyzed. The experiments were also conducted in the dark.

### Reduction in simplified suspension

 Silver nitrate (AgNO_3_) at 9.3 × 10^−3^−0.93 mM was allowed to react with POM at 9.0−143.2 mg C L^−1^ at pH 5.6−8.6. Modeling calculations confirmed that under all pH conditions >99.9% of the Ag was present as Ag^+^ (Visual MINTEQ 3.1). The suspensions were rotated at 500 rpm at 25 °C to ensure uniform light exposure and a well-mixed suspension^37^ in a photo-chemical reactor equipped with a water-circulating jacket for temperature control (XPA-7, Nanjing Xujiang Electromechanical Plant, China). The simulated sunlight was provided by a xenon source lamp (250−1100 nm) without light filters at 500 W/m^2^. AgNPs production was also evaluated under natural sunlight as well as in the dark (covered with aluminum foil). A parallel experiment was performed to study the effect of O_2_ on Ag^+^ reduction in which the suspension was purged with high-purity N_2_ for at least 30 min before exposure to simulated sunlight. SOD (150 U mL^−1^) was added to the suspension to determine the role of ${\mathrm{O}}_2^{\mathrm{\cdot-}}$. All experiments were conducted with at least duplicate samples.

### Characterization of AgNPs

At each time point, the suspensions were immediately filtered through a 0.45-μm filter and the resulting AgNPs in liquid phase were tracked by UV–Vis spectrophotometry at 300−800 nm. After rinsing with Milli-Q water, the POM was freeze-dried for X-ray Powder Diffraction (XRD, Ultima IV, Rigaku, Japan) and X-ray Photoelectron Spectrometer (XPS, ESCALAB 2500Xi, Thermo, USA) analyses. Transmission electron microscopy (TEM) with energy dispersive X-ray spectrometry (EDS) (JEM200CX, Japan) was performed at an accelerating voltage of 200 kV. NPs size was obtained using Nano Measure System 1.2.0 to analysis TEM images of at least 300 particles. Liquid chromatography inductively coupled plasma mass spectrometry (LC-ICP-MS) was also applied in the work to characterize the AgNPs at low concentrations^49^. Ultrafiltration coupled with inductively coupled plasma mass spectrometry (ICP-MS, Thermo-iCAP Q, USA) was used to quantitate Ag^+^ and AgNPs in liquid and particulate phase. The Ag^+^ in the liquid phase was measured by a 3-kDa centrifugal ultrafilter (Amicon Ultra-15 3 kDa, Millipore)^50,51^; the resulting AgNPs were then quantified by subtracting the Ag^+^ concentration from the total Ag concentration. The AgNPs on POM were extracted with 3 mL of 2.5 mM tetrasodium pyrophosphate (TSPP)^52^. The AgNPs were then quantified as described for the liquid phase. The results are presented as mean ± s.d. based on the results of *n* = 3 samples.

### FTIR, solid-state ^13^C NMR, and EPR analyses

To identify the structural components in POM responsible for Ag^+^ reduction, FTIR spectrometry (Nicolet iS10, Thermo, USA), solid-state ^13^C NMR (Burker Avance IIIHD 400 WB), and electron paramagnetic resonance spectrometry (EPR, EMX 10/12, Bruker, Germany) with a resonance frequency of 9.77 GHz of POM were performed^53^. The dimethyl sulfoxide (DMSO) and 5,5-dimethyl-1-pyrroline-N-oxide (DMPO, J&K Scientific Ltd, Shanghai, China) at 100 mM was used to trap the ${\mathrm{O}}_2^{\mathrm{\cdot-}}$ and generate the EPR signals (DMPO-${\mathrm{O}}_2^{\mathrm{\cdot-}}$) in the POM recorded in EPR spectra. A parallel experiment was also conducted using a DOM solution, released from POM, to rule out its potential effect on ${\mathrm{O}}_2^{\mathrm{\cdot-}}$ generation.

## Supplementary information

Supplementary Information

## Data Availability

The authors declare that the data supporting the findings of this study are available within the paper and its Supplementary Information files.
