# Supplementary material for: Significant contribution of metastable particulate organic matter to natural formation of silver nanoparticles in soils
Source: Nat Commun. 2019 Aug 21;10:3775. doi: 10.1038/s41467-019-11643-6 (PMC6704160; doi:10.1038/s41467-019-11643-6)
Supplement: Supplementary file 1 — Supplementary Information [file 41467_2019_11643_MOESM1_ESM.pdf]

Supplementary Information for

Significant contribution of metastable particulate organic matter to natural formation of silver nanoparticles in soils

Huang *et al*.

Supplementary figures

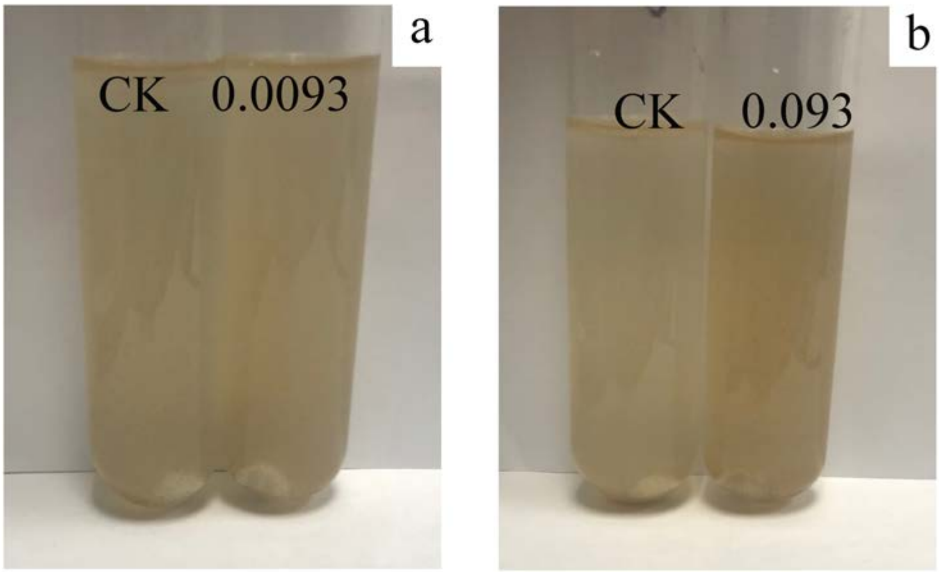

Supplementary Figure 1. Color evolution of samples with particulate organic matter suspension. (**a**) 9.3×10^{-3} and (**b**) 9.3×10^{-2} mM silver ions (Ag^+) were incubated with particulate organic matter (POM) at 143.2 mg C L^{-1} at pH 8.6 under natural sunlight for 24 h. No SPR absorbance intensity of the AgNPs appeared in the UV-vis spectra.

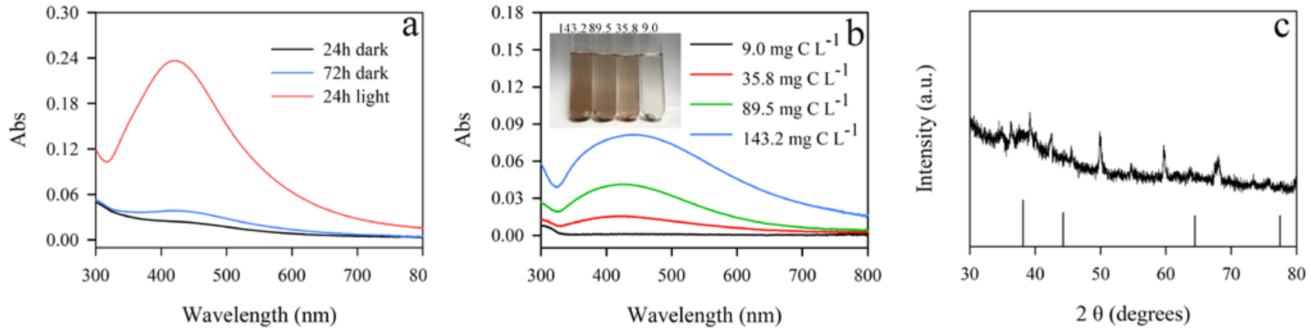

Supplementary Figure 2. Characteristics of silver nanoparticles in simplified suspension. Silver ions (Ag^+) at 0.93 mM was incubated with particulate organic matter (POM) suspension (9.0−143.2 mg C L^{-1}) at pH 8.6 at 25°C for 24 h. (**a**) UV-vis spectra of AgNPs with or without simulated sunlight and (**b**) under natural sunlight. (**c**) XRD analysis of particulate phase in the dark, with no diffraction peaks assignable to metallic Ag.

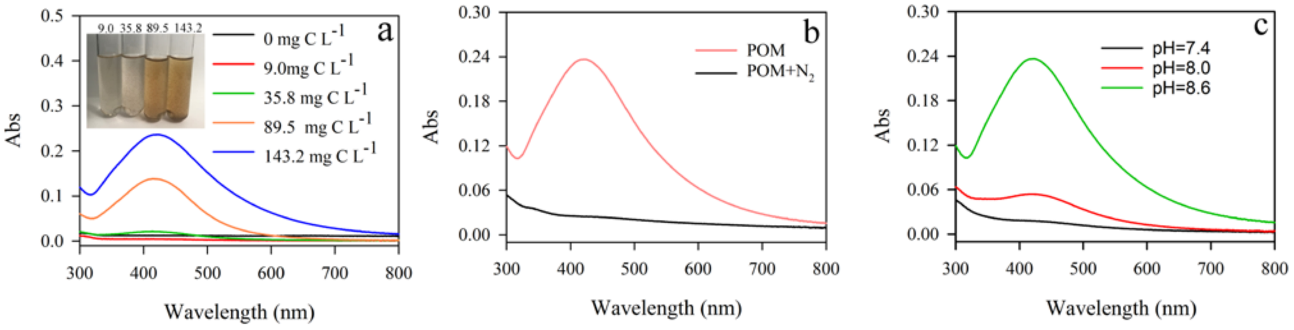

Supplementary Figure 3. Environmental factors modulate AgNPs formation. Formation of AgNPs under simulated sunlight at 25°C after 24 h as a function of (**a**) the particulate organic matter (POM) concentration (0−143.2 mg C L^{-1}), (**b**) the level of oxygen, and (**c**) pH.

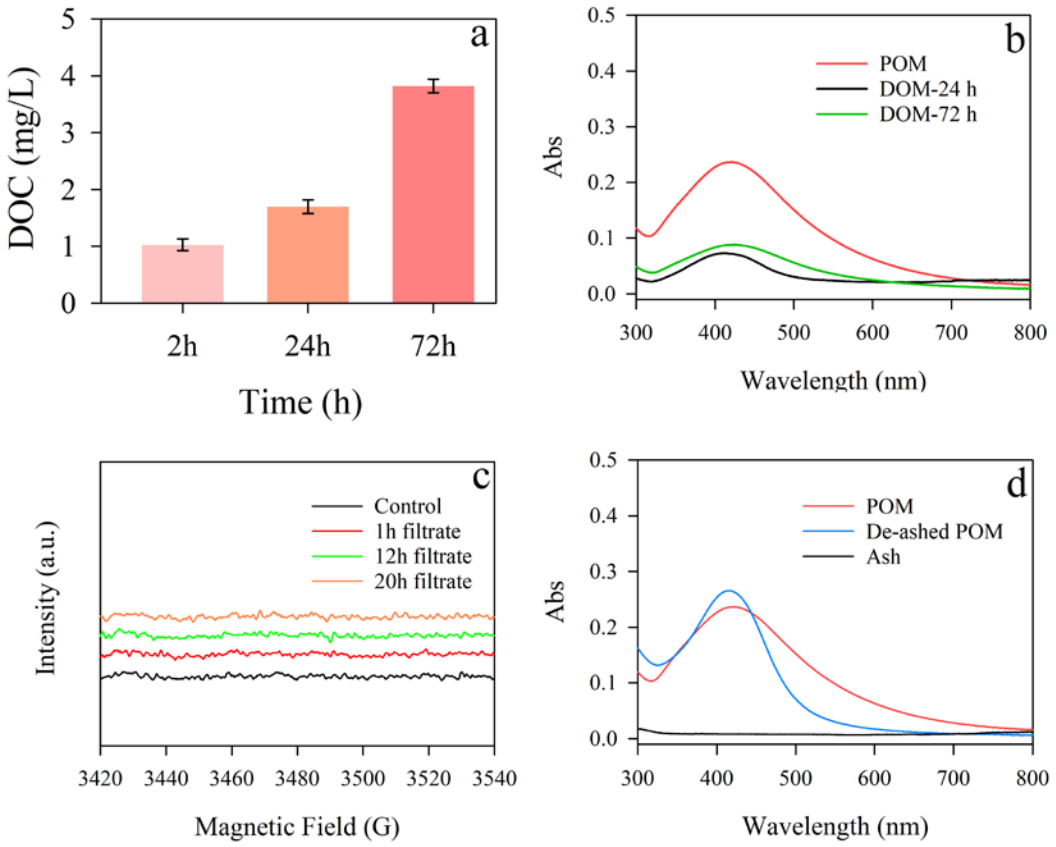

Supplementary Figure 4. Minimal role of dissolved organic matter and ash of POM on AgNP formation. (**a**) Kinetics of dissolved organic matter (DOM) released from particulate organic matter (POM) (143.2 mg C L^{-1}) at pH 8.6, with error bars representing the standard deviation of the mean of the triplicates. (**b**) Minimal effects of DOM on AgNP formation under simulated sunlight illumination for 24 h. (**c**) EPR spectra recorded for the DMPO-$O_2^{\cdot-}$ adduct in the released DOM at 1 h, 12 h, and 20 h after 1 h of irradiation, pH 8.6, and 25°C. (**d**) UV-vis spectra of AgNPs produced by the interaction of 0.93 mM silver ions (Ag^+) with 800 mg POM L^{-1} or 800 mg de-ashed POM L^{-1}, or 490 mg ash L^{-1} at pH 8.6 under simulated sunlight at 25°C for 24 h, with comparable SPR absorbance intensity between intact and de-ashed POM samples but no SPR absorbance in ash samples.

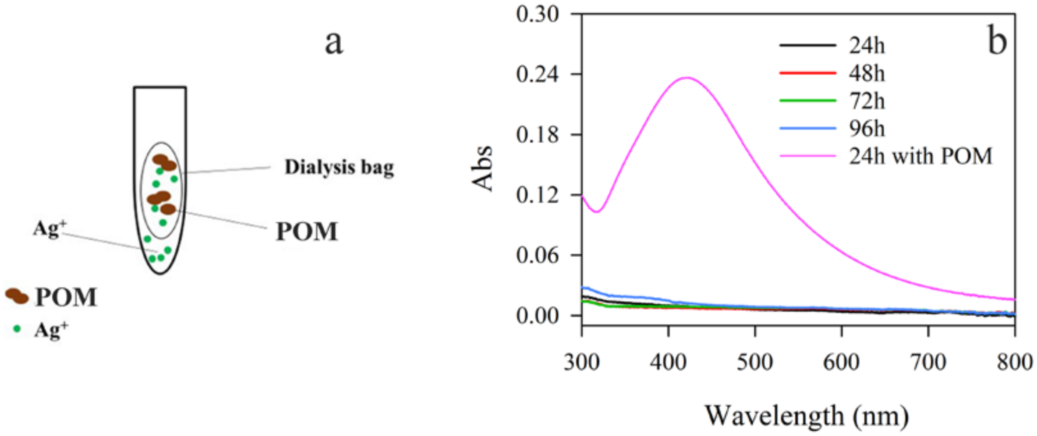

Supplementary Figure 5. Incubation of silver ions with POM in a dialysis bag. (**a**) Schematic illustrating that the dialysis bag (pore size < 1 nm; Spectrum, USA) allowed for direct contact between silver ions (Ag^+) and POM in 15 mM borate buffer at pH 8.6 under simulated sunlight and a temperature of 25°C, while Ag^+ in the bulk solution (i.e., outside the bag) was physically separated from the POM. (**b**) UV-vis spectra show that AgNPs were not observed in the bulk solution, without POM, but were present in the suspension in the dialysis bag.

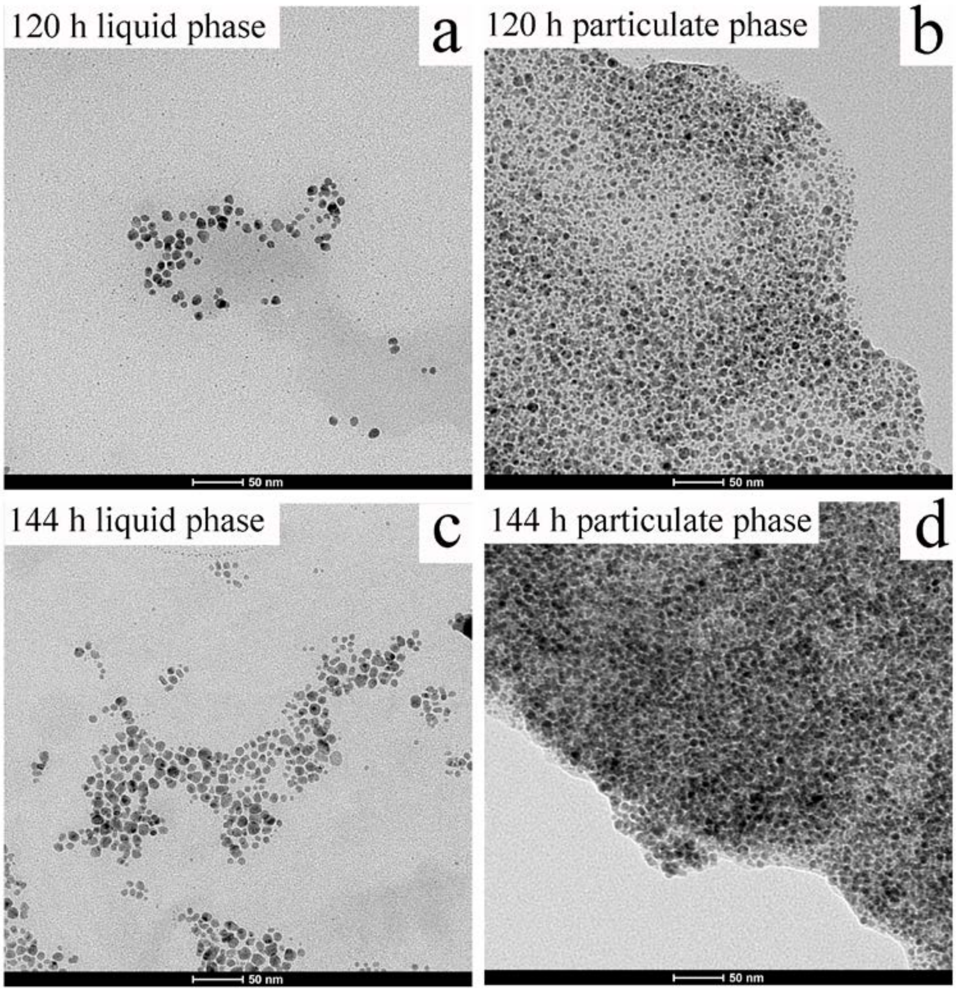

Supplementary Figure 6. TEM images of AgNPs in simplified suspension. TEM images of AgNPs in (**a**) liquid phase after 120 h, (**b**) in particulate phase after 120 h, (**c**) in liquid phase after 144 h, and (**d**) in particulate phase after 144 h reaction of 0.93 mM silver ions (Ag^+) with particulate organic matter (POM) at 143.2 mg C L^{-1} under simulated sunlight, pH 8.6, and a temperature of 25°C.

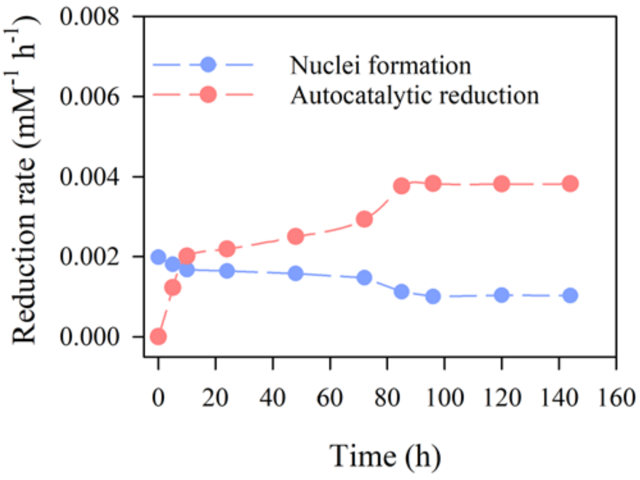

Supplementary Figure 7. Reduction rates of nuclei formation and autocatalytic surface reduction. Detailed information is given in the main text.

Supplementary Tables

Supplementary Table 1. Elemental composition and integrated solid-state ^{13}C NMR data for particulate organic matter (POM).

<table>
<tr><th rowspan="2">POM</th><th rowspan="2">Organic matter content (%)</th><th rowspan="2">Ash (%)</th><th rowspan="2">C[a]</th><th rowspan="2">H[a]</th><th rowspan="2">N[a]</th><th colspan="4">Chemical shift (ppm) and carbon assignment[b]</th></tr>
<tr><th>0–48 Aliphatic</th><th>48–109 Carbohydrate</th><th>109–140 Aromatic</th><th>140–162 Phenolic</th></tr>
<tr><td>POM</td><td>38.7</td><td>61.3</td><td>17.9±0.28</td><td>2.8±0.12</td><td>0.15±0.014</td><td rowspan="2">13.1</td><td rowspan="2">77.6</td><td rowspan="2">6.1</td><td rowspan="2">2.4</td></tr>
<tr><td>De-ashed POM</td><td>99.2</td><td>0.8</td><td>49.4±1.77</td><td>6.34±0.52</td><td>0.37±0.022</td></tr>
</table>

[a] Bulk element composition (%)

[b] Solid-state ^{13}C NMR data of POM

Supplementary methods

Supplementary method 1. Separation method of dissolved organic matter (DOM) released from particulate organic matter (POM) for the detection of $O_2^{\cdot-}$

To investigate the possible role of DOM released from POM on the production of $O_2^{\cdot-}$, a POM suspension (28.6 g C L^{-1}; 1.6 g POM in 10 ml borate buffer, pH 8.6) was stirred in the photo-chemical reactor without light for a period of time ranging from 0 to 20 h. At 1 h, 12 h, and 20 h, the suspension was collected and immediately passed through a 0.45-μm filter. The filtrates were analyzed for DOM released from POM (Note: a POM suspension of 1.4 g C L^{-1} was obtained by diluting 0.05 ml of a POM suspension containing 28.6 g C L^{-1} to a volume of 1 ml). The 0.05-ml filtrate was mixed with 0.9 ml of DMSO and exposed to light for 1 h; it was then spiked with 0.05 ml of 2 M DMPO. Thus, the solution at 15 mM borate buffer for $O_2^{\cdot-}$ detection by EPR contained the DOM released from POM (1.4 g C L^{-1}) and 100 mM DMPO.

Supplementary method 2. Effect of ash on the photoreduction of Ag^+

Ag^+ can be reduced on the surface of colloid silica and bentonite under alkaline conditions with or without light irradiation.[1, 2] To investigate the possible role of the minerals embedded in POM on Ag^+ reduction, de-ashed POM was prepared and used at a concentration of 800 mg L^{-1} in a reaction with 0.93 mM Ag^+ in 25 ml of 15 mM borate buffer, pH 8.6. The samples were irradiated by xenon light exposure (500 W m^{-2}) at 25°C for 24 h. To further demonstrate the ineffective role of inorganic matter in the reduction of Ag^+, the ash was collected after heating at 800°C for 5 h. Ash concentration used was 490 mg L^{-1}, based on the ash proportion within POM (Supplementary Table S1), which was tested in a photo-chemical reactor under the same conditions as described for the de-ashed POM samples.

Supplementary references

1. Kónya, J.; Nagy, N. M.; Földvári, M. The formation and production of nano and micro particles on clays under environmental-like conditions. *J Therm Anal Calorim* 2005, 79 (3), 537-543 (2015).

2. Praus, P. Study of silver adsorption on montmorillonite. *J Brazil Chem Soc* 2008, 19 (3), 549-556 (2008).
